# Arbuscular mycorrhizal fungi negatively affect soil seed bank viability

**DOI:** 10.1002/ece3.2491

**Published:** 2016-10-05

**Authors:** Mahmood Maighal, Mohamed Salem, Josef Kohler, Matthias C. Rillig

**Affiliations:** ^1^ Institut für Biologie Plant Ecology Freie Universität Berlin Berlin Germany; ^2^ Berlin‐Brandenburg Institute of Advanced Biodiversity Research (BBIB) Berlin Germany

**Keywords:** arbuscular mycorrhiza, fungi, plant–soil (belowground) interactions, root exclusion compartments, seed viability, soil seed bank, symbiosis

## Abstract

Seed banks represent a reservoir of propagules important for understanding plant population dynamics. Seed viability in soil depends on soil abiotic conditions, seed species, and soil biota. Compared to the vast amount of data on plant growth effects, next to nothing is known about how arbuscular mycorrhizal fungi (AMF) could influence viability of seeds in the soil seed bank. To test whether AMF could influence seed bank viability, we conducted three two‐factorial experiments using seeds of three herbaceous plant species (*Taraxacum officinale, Dactylis glomerata*, and *Centaurea nigra*) under mesocosm (experiments 1 and 2) and field conditions (experiment 3) and modifying the factor AMF presence (yes and no). To allow only hyphae to grow in and to prevent root penetration, paired root exclusion compartments (RECs) were used in experiments 2 and 3, which were either rotated (interrupted mycelium connection) or kept static (allows mycorrhizal connection). After harvesting, seed viability, soil water content, soil phosphorus availability, soil pH, and hyphal length in RECs were measured. In experiment 1, we used inoculation or not with the AMF *Rhizophagus irregularis* to establish the mycorrhizal treatment levels. A significant negative effect of mycorrhizal hyphae on viability of seeds was observed in experiments 1 and 3, and a similar trend in experiment 2. All three experiments showed that water content, soil pH, and AMF extraradical hyphal lengths were increased in the presence of AMF, but available P was decreased significantly. Viability of seeds in the soil seed bank correlated negatively with water content, soil pH, and AMF extraradical hyphal lengths and positively with soil P availability. Our results suggest that AMF can have a negative impact on soil seed viability, which is in contrast to the often‐documented positive effects on plant growth. Such effects must now be included in our conceptual models of the AM symbiosis.

## 1 Introduction

Arbuscular mycorrhizal (AM) fungi are a key component of the soil ecosystem, especially in grasslands (Smith & Read, 2008). They provide numerous services to plants, including enhanced nutrient uptake (particularly P), or increased plant resistance against pathogens and abiotic stressors (Smith & Read, 2008). AM fungi also have an impact on plant diversity patterns in a variety of ecosystems (Van der Heijden et al., 1998; Hartnett & Wilson, 1999), for example, by providing differential benefits to members of the plant community. Mycorrhizal plant growth responses range from positive to negative, suggesting that mycorrhizae operate along a mutualism–parasitism continuum, depending on the relative benefits and costs of the symbiosis (Johnson, Graham, & Smith, 1997; Johnson & Graham, 2013); such effects may differ for different plant life history stages (Varga, 2015).

Effects of AM fungi on plant growth are very well documented (Smith & Read, 2008), but virtually nothing is known about their influence on the seed bank, most likely because this is a plant life history stage generally viewed not to be influenced by AM fungal colonization. In general, the early stages of plants appear to be neglected with respect to effects of arbuscular mycorrhiza. Recently, Varga (2015) showed that AM fungi can negatively influence seed germination while still improving plant growth subsequently. Such early‐stage effects are important in terms of understanding the net effects of the AM symbiosis on plants. Thus, there is a pressing need to know whether AM fungi can influence plant seeds and the soil seed bank.

The soil seed bank comprises all viable seeds present on‐or‐in the soil or in the associated litter (Simpson, Leck, & Parker, 1989). Present in nearly all terrestrial ecosystems (Baker, 1989), the seed bank plays a prominent role in the ecology of many plant species (Roberts, 1981; Thompson, 1987; Leck, Parker, & Simpson, 1989; Thompson, Bakker, & Bekker, 1997; Baskin & Baskin, 1998). Seeds can remain viable in the soil for different periods of time depending on plant species and soil conditions (Priestley, 1986; Thompson, 1993; Buhler & Hartzler, 2001; Poschlod, Tackenberg, & Bonn, 2005; Conn, Beattie, & Blanchard, 2006). The soil seed bank plays an important role in the composition and succession of many plant communities (Kemp, 1989; Thompson, 1992; Van der Valk, 1981). Seed banks can be an important component for understanding the dynamics of plant populations, communities, and ecosystem functioning (Silvertown, 1982; Kalisz, 1991; Kalisz & McPeek, 1992; Günter, 1997; Bekker et al., 1998; Cabin, Mitchell, & Marshall, 1998). Persistent seeds in the soil seed bank can also represent a reserve of genetic potential accumulating overtime (Simpson et al., 1989).

Soil organisms other than AM fungi can have a direct effect on the soil seed bank, and such effects have been documented. For example, seeds may be affected by the activity of soil biota, such as the transfer and burial of seeds by earthworms (Grant, 1983; Van der Reest & Rogaar, 1988; Thompson, Green, & Jewels, 1994) or other soil animals (Grant, 1983; Shumway & Koide, 1994; Willems & Huijsmans, 1994; Bernhardt, 1995). Furthermore, fungal pathogens are a main cause of mortality of buried seeds (Leishman, Masters, Clarke, & Brown, 2000), and abiotic conditions, such as soil moisture, moderate their effect on seeds (Schafer & Kotanen, 2003).

Given the already documented effect of other soil biota on the seed bank, the main goal of this research was to explore whether and how AM fungal mycelium could influence the seed bank, and specifically seed viability. To address this goal, we carried out three experiments in the glasshouse and in the field, using buried seeds of three grassland species. For all studies here, we used seeds that we added to soil experimentally, rather than using an in situ soil seed bank, because for the latter, past effects of AM fungi are impossible to exclude.

## 2 Materials and Methods

### 2.1 Seeds and soil

In all our experiments, seeds of three herbaceous plant species (*Taraxacum officinale* G. H. Weber ex Wiggers)*, Dactylis glomerata* L., and *Centaurea nigra* L. were used; these were obtained from a commercial supplier (Albert Treppens & Co Samen GmbH, Berlin, Germany). We chose these species because their seeds do not germinate when buried in soil at a temperature generally permitting fungal growth (Mitschunas, Wagner, & Filser, 2006), and they occurred at‐or‐near the site from which soil for glasshouse experiments was obtained (see below). Seeds of *C. nigra* generally had quite high viability, whereas seeds of the other two species had low viability in preliminary trials; as the direction of a potential effect of AM fungi is not clear a priori, we thus also represented different inherent seed viabilities.

The soil used in the glasshouse experiments was an Albic Luvisol from a meadow in Dahlem (Berlin, Germany). It was a fresh loamy and sandy soil having the following properties: N = 0.12%, C = 1.87%, 74% sand, 18% silt, and 8% clay and the soil pH was 7.1 (Rillig et al., 2010). The soil was obtained at a depth of 10‐40 cm below the surface, then air‐dried and passed through a 2‐cm sieve to remove plant material and stones, and to homogenize it. We chose this soil due to its high AM inoculum potential (Rillig et al., 2010).

### 2.2 Preparation of root exclusion compartments

Two of the three experiments were carried out in the glasshouse and one (experiment 3) was set up in the field. A modified ingrowth core design (Johnson, Leake, & Read, 2001) was used for experiments 2 and 3 only. Paired root exclusion compartments (RECs) were used in experiments 2 and 3, which were either rotated (interrupted mycelium connection) or kept static (mycorrhizal connection intact), thus providing a soil volume with or without AM fungal mycelium in which to place seeds.

The RECs (diameter 3 cm, height 12 cm) were prepared by covering the sides and bottom of the core with 30‐μm‐nylon mesh (Sefar Nitex 03‐30/18; Sefar GmbH, Edling, Germany) in order to allow only hyphae to grow in and to prevent root penetration. The RECs were filled at the beginning of the experiment with nonsterilized soil (see above).

### 2.3 Experiments

A series of three experiments, described below, were performed with the aim to explore the effects of AM fungal mycelium on the viability of seeds in the soil seed bank. Each experiment had a two‐factorial design, where each treatment was replicated ten times. The first factor was species identity, consisting of three species of plants (*T. officinale, D. glomerata*, and *C. nigra*). The second factor was presence‐or‐not of AM fungi with two levels (without and with AM fungal mycelium); this was achieved in experiments 2 and 3 with the REC arrays. Half of the RECs were kept static after placing them in the soil with the purpose to allow hyphal ingrowth, and the other half were rotated by 1–2 mm three times a week around their vertical axes in order to sever any hyphae crossing the mesh barrier. We previously showed that in the same soil, rotating cores for excluding AM fungi had no confounding effects on soil abiotic properties (Leifheit, Verbruggen, & Rillig, 2014).

Experiments were set up under glasshouse conditions (experiments 1 and 2; 12 hr of light; 20°C/18°C temperature day/night; 45% relative humidity) and field conditions (experiment 3). Fifty seeds of each species were enclosed in plastic mesh bags (2×2 cm, mesh pore size 500 μm) to protect them from seed predators and facilitate harvest at the end of the experiment. The mesh bags were placed inside the RECs equidistantly (2±1 mm, distance of mesh bag from side of core; 5±1 cm deep from the surface). We selected this depth because it is a reasonable depth for the presence of viable seeds in the soil seed bank and mycorrhizal fungi in soil. As host plants for the mycorrhizal network in the pot experiments, we used *Trifolium repens* in experiment 1 and Sudangrass (*Sorghum x drummondii*) in experiment 2. Both species are frequently used in mycorrhizal studies. Seeds of these host plants were sown on sterile wet paper in plastic containers in a climate chamber at 20°C and 16 hr of light. Seedlings were then transplanted four weeks after germination into the experimental pots.

#### 2.3.1 Experiment 1: Glasshouse inoculation‐based study

In this two‐factorial experiment, the first factor (seed species identity) consisted of three different seed species while the second factor was the addition of AM fungi with two levels (without and with AM fungi). Half of the pots (3 L) were filled with autoclaved soil (to eliminate any AM fungal propagules), mixed with 10 g mycorrhizal pellets (AM fungi treatment); containing the AM fungus *Rhizophagus irregularis* (Blaszk, Wubet, Renker & Buscot) C.Walker & Schuessler (formerly *Glomus intraradices*) (Biomyc^®^ Brandenburg, Germany). The other half of the pots received the same autoclaved soil but with autoclaved pellets for the nonmycorrhizal control (no AM fungi treatment); a microbial wash was prepared and added to all pots as described by Achatz et al., (2014).

#### 2.3.2 Experiment 2: Glasshouse study using rotated RECs

For confirming the results of experiment 1 and to eliminate the possibility that results were driven by autoclaved soil and a single added AM fungal species, we carried out another experiment with a rotated REC design. This two‐factorial experiment with species identity and AM fungal mycelium presence as factors was carried out in the glasshouse. AM fungal mycelium presence consisted of the levels rotated (interrupted mycelium connections) or kept static (AM fungal mycelium present inside RECs). Each pot (3 L) at the beginning of the experiment was filled with nonsterilized field soil containing an AM fungal community.

#### 2.3.3 Experiment 3: Field study using rotated RECs

This experiment was conducted in the field with a seminatural plant community, consisting predominantly of *Lolium perenne* and *Poa annua,* during April to June 2013 at experimental garden plots of Freie Universität Berlin; this general site was used in a previous experiment using RECs (Achatz & Rillig, 2014). We used nonsterilized soil inside the RECs; we filled into the RECs the same soil as in the pot experiments. Twenty RECs were placed in the field, always with a distance of 5 cm between the cores. To enable a connection to the existing mycorrhizal network in the field plot, half of the compartments were kept static after placing them in the soil (depth: ca. 12 cm), the others were rotated three times per week by 1‐2 mm severing the hyphae attempting to cross into the RECs (Achatz et al., 2014). Fifteen weeks after planting, the seeds were taken out of the RECs and a soil sample from each REC was taken for further analysis.

### 2.4 Postharvest measurements

All measurements were carried out with soil from RECs (experiments 2 and 3), or the experimental soils in pots (experiment 1). In order to determine the available phosphorus (P) content in the soil, the calcium–acetate–lactate‐soluble phosphorus content was determined spectrophotometrically according to the German standard method DIN 3.4.1.30.2a (Blume, Deller, & Leschber, 2000). Soil pH was assessed at the end of the experiment with a pH meter (Knick 761 Calimatic) in a 1:5 (w/v) aqueous dilution. Soil water content was determined as weight loss after drying at 70^**°**^C for 72 hr.

Hyphal length of AM fungi was determined in 4.0 g of fresh soil by an aqueous extraction and membrane filter technique modified after Jakobsen, Abbott, and Robson (1992). Hyphae of AM fungi were distinguished microscopically at (200×) from other fungal hyphae as described by Rillig, Field, and Allen (1999).

Seeds were extracted from the RECs or soils in pots. Fifty seeds of every species per experimental unit were counted and tested by the modified method of Malone (1967) and staining them with a solution of 2,3,5‐Triphenyltetrazolium chloride (TTC; Sigma‐Aldrich, St. Louis). The dicotyledonous species, (*C. nigra, T. officinale*) and the grass (*D. glomerata*) were exposed to 0.1% and 1% solution of TTC, respectively. After keeping the seeds in darkness for 48 hr at 20°C and rinsing five times in sterile distilled water, the seeds were agitated between cover slides to remove the seed coat (testa) and then observed using a light microscope. Embryos which were completely pink to red were considered viable, while those embryos which were partially white, yellow, or brown were categorized as not viable (Van Waes & Deberg, 1986).

### 2.5 Statistical analysis

Seed survival data were analyzed in R (version 2.14.1) through mixed‐effects generalized linear models. We used the function (*glmer)* in the package lme4 for this purpose (Zuur, Ieno, Walker, Saveliev, & Smith, 2009). Errors were assumed to follow a binomial distribution. In all three experiments, we used mycorrhizal status and plant species as categorical predictors and we considered their interaction. Block effects were accounted through a random effects factor. In experiments 1 and 2, we assumed each pot to be a different block. In experiment 3, each neighboring REC pair (rotated and nonrotated RECs) was a different block.

For pH, hyphal length, and available phosphorus, we implemented two‐way ANOVAs with the same predictors as for seed survivorship. Data on soil pH, hyphal length, and available P in soil were log‐transformed and seed survival were arcsine‐transformed as necessary to meet the assumptions of normality and homoscedasticity.

Differences between the hyphal connection/presence treatments were analyzed by single factor ANOVA including all the data. We used Tukey–Kramer HSD to conduct multiple comparison tests. The relationships among hyphal length, water content, seed viability, soil P concentrations, and soil pH were tested via Pearson correlation coefficients.

## 3 Results

### 3.1 Demonstration of treatment effectiveness

**Figure 1:**
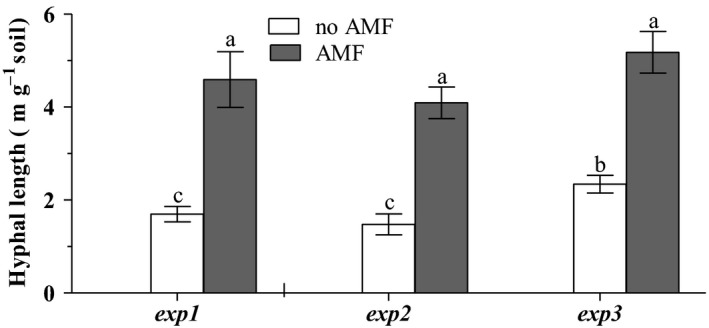
Demonstration of treatment effectiveness. Effects of RECs (no AMF) and static core (AMF) on hyphal length of AM fungi in soil in all experiments. Means and standard deviation (*n* = 10) are shown. Different letters indicate significant differences between the treatments at *p* < .05 according to the Tukey–Kramer HSD test. [Correction added on 25 October 2016, Figure 1 is now corrected in this version.]

In all three experiments, irrespective of field or glasshouse or RECs or inoculation‐based approaches, we found significant differences in AM fungal hyphal abundance between the AMF and no AMF treatments (Figure 1). Hyphal abundances were always clearly higher in the AMF treatments.

### 3.2 Effect of AM fungi on seed viability

**Figure 2:**
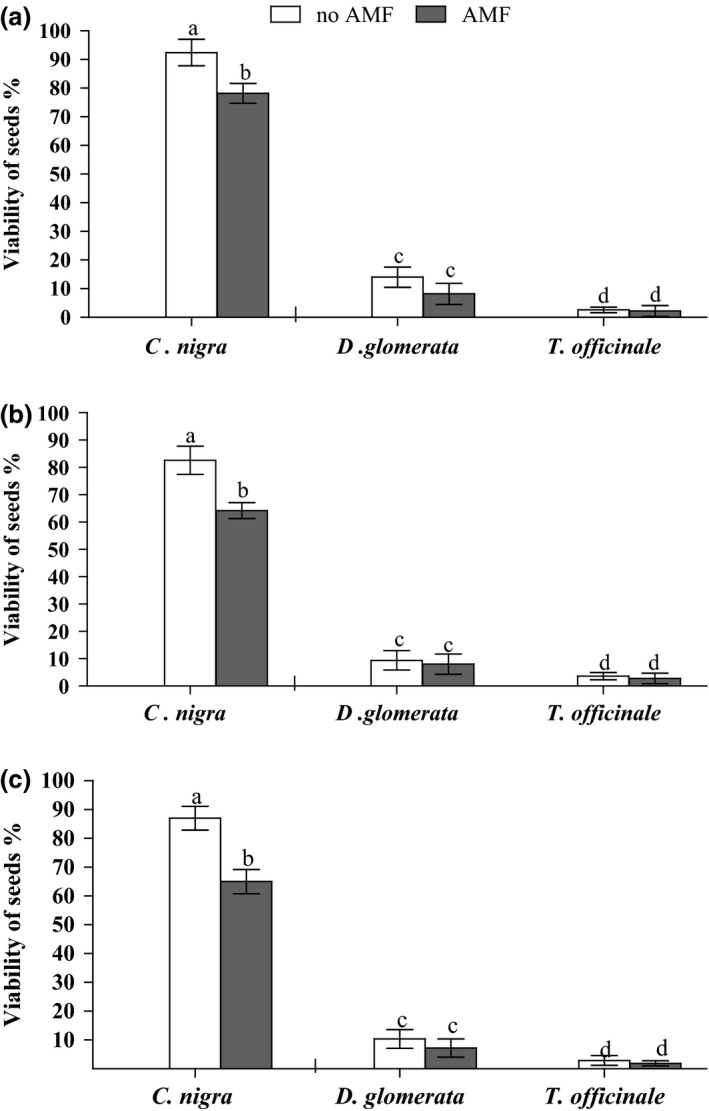
Effects of AM fungi on seed viability (%) of *C. nigra, D. glomerata,* and *T. officinale*. AM fungi presence was either achieved (a) by adding inoculum to an autoclaved soil in experiment 1; (b) using rotated/static RECs in the glasshouse (experiment 2); or (c) with rotated/static RECs in field plots (experiment 3). Means and standard deviation (*n *= 10) are shown. Different letters indicate significant differences between the treatments at *p *< .05 according to the Tukey–Kramer HSD test

In our experiments, we investigated the impact of AM abundance on seed viability. We found significant main effects for the factor “mycorrhiza” and the factor “seed species” in all three experiments (Table 1), with the interaction term significant in experiments 2 and 3, but not in experiment 1. There were consistently negative effects of AM fungal presence on seed viability of *C. nigra* in all three experiments*,* but there were no such effects for seeds of *T. officinale* and *D. glomerata* in any experiment (Figure 2). Overall seed viability, irrespective of treatment was much lower for *T. officinale* and *D. glomerata* than for *C. nigra* in all three experiments (Table 1, Figure 2).

**Table 1:** F values (ANOVA) for the effects of AM fungi (AMF) and seed species, and their interaction on viability of seeds of three species (***p < .001) (*n* = 10)

Experiment	AMF	Species	AMF × species
Experiment 1	52.80***	1.41***	4.81
Experiment 2	137.83***	2.55***	24.45***
Experiment 3	183.21***	5.33***	106.66***

In experiment 1, the AMF treatment was achieved by inoculation or not inoculating, whereas in the experiments 2 and 3, this was achieved using rotated/static RECs.

### 3.3 Soil properties

**Figure 3:**
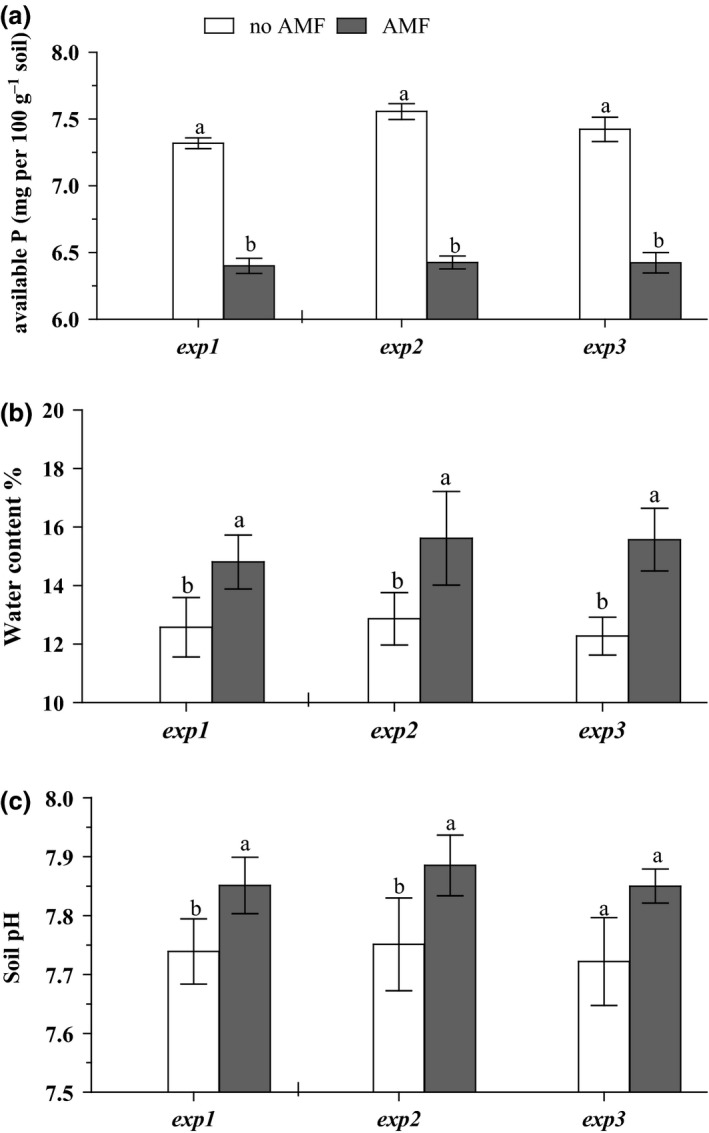
Effects of rotated RECs (no AMF) and static RECs (with AMF access) on (a) phosphorus concentration of soil, (b) water content, and (c) soil pH for all experiments. Means and standard deviation (*n *= 10) are shown. Different letters indicate significant differences between the treatments at *p *< .05 according to the Tukey–Kramer HSD test

We assessed the impact of AM fungi on soil characteristics to gain insight into potential AM fungal‐mediated effects on seed viability. We found that AM fungi had a significantly negative effect on available P content in soil as compared to the control (Figure 3). In addition, we found that water content and soil pH had significantly increased with AM fungi compared to the control (without AMF) (Figure 3). In the field experiment, seed viability was negatively related with soil AM fungal hyphal length, pH, and water content, but positively with soil P (Table 2).

**Table 2:** Pearson correlation coefficients for all variables measured in the field experiment (*p < .05; **p < .01) (*n* = 10)

	Viability of seeds	Hyphal length	Soil pH	Water content
Viability of seeds	–	–	–	–
Hyphal length	−0.657**	–	–	–
Soil pH	−0.616*	NS	–	–
Water content	−0.714**	0.549*	NS	–
Phosphorus	0.803**	−0.692**	0.773**	−0.564*

## 4 Discussion

We showed through our three complementary experiments, which employed different means of manipulating AM fungal abundance, and which were carried out in the field and in pots, that AM fungi had a clear and negative impact on soil seed viability for one of the three species of plants we examined. Even though effects were not significant for seeds from the other two species, there was a similar trend, suggesting that effects may be more general. The fact that this result was robust to the particularities of experimental design, each of which has its advantages and drawbacks, increases confidence in our findings. For example, in one case (experiment 1), only one AM fungal species was involved (added as inoculum), whereas in the other experiments, communities of AM fungi were likely active. Importantly, we observed this effect in the field as well as in pots.

As we assumed that AM fungi would be unlikely to directly affect seed viability, we measured a number of soil parameters known to influence soil seed viability, which could also be influenced by AM fungal hyphae. Seed viability can be affected by soil physicochemical properties (Pakeman, Small, & Torvell, 2012), such as soil pH and soil water content (Bekker et al., 1998; Wagner & Mitschunas, 2008), and perhaps nutrients. Other factors include the soil microbial community (Schafer & Kotanen, 2003; Dalling, Davis, Schutte, & Arnold, 2011), which could in turn be influenced by the soil physicochemical parameters. For example, soil water content can affect the viability of seeds in the soil both directly and indirectly due to its interrelation with other parameters such as aeration and temperature. Soil moisture potentially affects germination of fungal spores and growth of soil fungi (parasitic or saprobic) colonizing seeds, in addition to affecting change in the soil microbial community, which may affect seed viability (Wagner & Mitschunas, 2008).

In this study, we found a close relationship between the increase in (local) soil water content, affected by the AM fungal treatment, and decrease in seed vitality. Perhaps the reason for this relates to water transport along AM fungi hyphae (Querejeta, Egerton‐Warburton, & Allen, 2003) into the compartment containing the seeds or perhaps the effect is due to effects on water content due to potential AM hypha‐mediated effects on soil aggregation (Rillig & Mummey, 2006). Another possibility is that allelochemical compounds were translocated to the seeds along the AM fungal hyphae, as has been shown to occur in the same experimental soils used here for other plant species (Achatz et al., 2014). Irrespective of the mechanism, which our study was not designed to disentangle, the higher water content could then have facilitated microbial growth, leading to the degradation of seeds.

AM fungi are functionally mostly associated with an increased uptake of phosphorus from the soil, but other nutrients can also be taken up and transported to the plant host (Smith & Smith, 2011). Our results accordingly showed decreased soil P availability with AM fungal presence in all three experiments (Figure 3). This decreased phosphorus in the soil, perhaps, also contributed to decreased seed viability, perhaps via effects on the soil microbial community. Data on P‐mediated effects on seed banks are sparse and do not permit isolation of P as the sole causal factor. However, available data agree with our findings. For example, Van der Valk and Rosburg (1997) collected seed bank samples in the northern Everglades along a phosphorus gradient with three vegetation zones, where they found the highest seed numbers in the zone with the highest available P.

Our results, besides adding novel, basic data on AM fungal effects on an important plant life history stage, could also have applied relevance, for example, in restoration. The seeds of desirable species could be rare and seeds of less desirable exotic species could be very abundant in the seed bank (St. John, 1998); in the beginning of the restoration process, AM fungi may confer an advantage to certain seed types by inhibiting viability of others. Harnessing such relationships could thus aid in encouraging successional trajectories through the addition or management of mycorrhizal inoculum, for example, by helping to control weeds (Jordan, Zhang, & Huerd, 2000). Future research should explore longer‐term experimentation with seed banks in the field to corroborate these findings.

## 5 Conclusion

Our results suggest that AM fungi can have a negative impact on soil seed viability, which is in contrast to the often‐documented positive effects on plant growth. This result highlights how symbionts may have different or even contrasting effects on different life history stages of their host; such data are important for estimating the net effects of the symbiosis across the entire life cycle of plants. These results invite further investigations on the generality of this finding in other plant species and ecosystems, and our findings should be included in our conceptual models of AM fungal effects on plant populations and communities.

## Conflict of interest

None declared.
